# Rapid screening and early precautions for carbapenem-resistant *Acinetobacter baumannii* carriers decreased nosocomial transmission in hospital settings: a quasi-experimental study

**DOI:** 10.1186/s13756-019-0564-9

**Published:** 2019-06-27

**Authors:** Norihisa Yamamoto, Shigeto Hamaguchi, Yukihiro Akeda, Pitak Santanirand, Narong Chaihongsa, Suntariya Sirichot, Suwichak Chiaranaicharoen, Hideharu Hagiya, Kouji Yamamoto, Anusak Kerdsin, Kazuhisa Okada, Hisao Yoshida, Shigeyuki Hamada, Kazunori Oishi, Kumthorn Malathum, Kazunori Tomono

**Affiliations:** 1 Department of Infection Control and Prevention, Osaka University Graduate School of Medicine, 2-2 Yamadaoka, Suita, Osaka, 565-0871 Japan; 2 Research Institute for Microbial Diseases, Osaka University, Osaka, Japan; 3 Faculty of Medicine Ramathibodi Hospital, Mahidol University, Bangkok, Thailand; 4 Department of Medical Statistics, Graduate School of Medicine, Osaka City University, Osaka, Japan; 5 Faculty of Public Health, Kasetsart University, Sakon Nakhon, Thailand; 6 Infectious Disease Surveillance Center, National Institute of Infectious Diseases, Tokyo, Japan

**Keywords:** Carbapenem-resistant *Acinetobacter baumannii* (CRAB), Nosocomial transmission, LAMP (Loop-mediated isothermal amplification), Rapid molecular diagnosis., Rapid intervention, Intensive care unit.

## Abstract

**Background:**

Active surveillance has the potential to prevent nosocomial transmission of carbapenem-resistant *Acinetobacter baumannii* (CRAB). We assessed whether rapid diagnosis using clinical specimen-direct loop-mediated isothermal amplification (LAMP), a rapid molecular diagnostic assay, and subsequent intervention, could reduce CRAB nosocomial transmission in intensive care units (ICUs).

**Methods:**

A before and after (quasi-experimental) study was conducted in two ICUs at the Mahidol University Faculty of Medicine Ramathibodi Hospital with 3 months of observational period followed by 9 months of interventional period. All patients were screened for CRAB using both the culture and LAMP method from rectal swab and/or bronchial aspirates (intubated patients only) upon admission, weekly thereafter, and upon discharge. During the pre-intervention period, we performed contact precautions based on culture results. In contrast, during the intervention period, we initiated contact precautions within a few hours after sample collection on the basis of LAMP results.

**Results:**

A total of 1335 patients were admitted to the ICUs, of which 866 patients (pre-intervention period: 187; intervention period: 679) were eligible for this study. Incidence rate of CRAB infection decreased to 20.9 per 1000 patient-days in the intervention period from 35.2 in the pre-intervention period (*P* < 0.02). The calculated hazard ratio of CRAB transmission was 0.65 (95% confidence interval [CI], 0.44–0.97). Risk factors for CRAB acquisition included exposure to carbapenem (hazard ratio, 2.54 [95% CI: 1.61–5.57]).

**Conclusions:**

LAMP screening for CRAB upon ICU admission proved feasible for routine clinical practice. Rapid screening using LAMP followed by early intervention may reduce CRAB transmission rates in ICUs when compared to conventional intervention.

**Electronic supplementary material:**

The online version of this article (10.1186/s13756-019-0564-9) contains supplementary material, which is available to authorized users.

## Background

*Acinetobacter baumannii* is an important opportunistic pathogen that causes a variety of nosocomial infections, especially in an intensive care unit (ICU) [1–4]. The organism frequently exhibits resistance to most commercially available antibiotics [1]. In particular, carbapenem-resistant *Acinetobacter baumannii* (CRAB) infections present limited therapeutic options and are associated with high morbidity and mortality as well as longer hospitalization times [3–6]. Patients colonized with CRAB have a greater risk of developing active CRAB infections as well as initiating nosocomial transmission [5, 6]. These risks from CRAB colonization motivate active surveillance and appropriate infection control measures against infected and colonized patients [3, 5, 7, 8].

Conventional methods to screen for CRAB do not sufficiently satisfy the requirements for a robust surveillance tool. Phenotypic methods generally require a few days to determine the antibiotic spectrum, resulting in delays in initiating preventive measures for carriers [9, 10]. Furthermore, the sensitivity is insufficient to identify colonized patients thoroughly [7, 11]. Molecular methods such as PCR could solve these disadvantages, but these techniques are generally laborious and expensive. Here, we establish a clinical specimen-direct Loop-mediated isothermal amplification (LAMP) assay for CRAB as an easier and more cost-effective molecular diagnostic system; isothermal amplification finishes within 20 min and costs are less than 3 U.S. dollars per sample [12]. Since *bla*_OXA-23_ is one of the most prevalent resistance mechanisms among CRAB in Asia, including Thailand which was 94.2% in our previous data in Bangkok, we targeted this genotype for our assay development [1, 13–15]. Our clinical specimen-direct LAMP obtains results within 40 min from sample collection to facilitate early isolation of positive patients. We proved its efficacy as a surveillance tool in an ICU where CRAB is especially problematic [12].

Importantly, active CRAB surveillance has been reported to reduce nosocomial transmission, especially during outbreak situations [3, 16], and several guidelines recommended its implementation [5, 17]. It is hypothesized that rapid detection of asymptomatic CRAB carriers enabled medical workers to implement preventive procedures earlier, thus reducing further transmission of CRAB when compared with the conventional culture method. The present study was divided into two arms to compare the incidence rate of transmission; a pre-intervention period and an intervention period. During the pre-intervention period, preventive measures were implemented following results of the conventional culture method while preventative measures were instituted by the results of LAMP during an intervention period. The primary aim of this study was to analyze whether rapid preventive screening of CRAB carriers reduces nosocomial transmission rates in ICU settings. We also sought to identify any risk factors associated with CRAB acquisition.

## Methods

### Study settings

This before and after (quasi-experimental) study was conducted in Mahidol University Faculty of Medicine Ramathibodi Hospital. The study was performed in two discrete medical and surgical ICUs, which had 12 and 15 beds respectively, between December 2013 and January 2015. Conventional culture screening was performed in the first three months (December 2013 to February 2014; pre-intervention period) and the clinical specimen-direct LAMP screening was applied in the subsequent nine months (May 2014 to January 2015; intervention period) following a 2-month wash-out period. All the patients entering the ICUs were screened for CRAB on admission; patients who did not carry CRAB, and those who stayed more than two days, were included due to the definition of nosocomial transmission [18]. The characteristics of the patients were recorded categorically. Circulatory diseased included heart failure, coronary artery disease and etc. Chronic kidney disease (CKD) included all individuals with an estimated glomerular filtration rate of less than 60 ml/min/1.73m^2^ on at least 2 occasions 90 days apart. Gastrointestinal disease included ileus, colitis, hemorrhoids, Crohn’s disease, and etc. Chronic respiratory disease included chronic obstructive pulmonary disease, asthma, other inflammatory respiratory disorders and etc.

### Procedures in the wards

Surveillance samples were obtained within 24 h of admission, weekly thereafter and on discharge. Specimens included rectal swabs and, if mechanically ventilated, bronchial aspirates were also obtained since those sites were reported to be appropriate for screening for CRAB [6, 17]. When either patient sample was positive, we defined the patient as a CRAB carrier. Full-time infection control nurses were assigned to each ward and were engaged in sampling specimens, collecting data, and managing contact precautions. All CRAB carriers were marked with cards to inform medical workers on the necessity of appropriate precautions. Cohorting included the use of a disposable gown and glove as well as isolation of patients using curtains. With the exception of starting time of precautions, we performed the same procedures in both periods. We collected potential confounding factors for all patients.

### Procedures in the laboratory

All clinical specimens were analyzed using both the conventional culture method and our LAMP assay method. Specimens collected from patients were cultured for at least 18 h and analyzed by conventional biochemical testing [11] as well as by MALDI-TOF mass spectrometry (Bruker, Leipzig, Germany) [19]. The Sensititre (Thermo Fisher Scientific, Cleveland, OH) with the THANF panel of antimicrobial agents was used for the susceptibility tests. Antimicrobial sensitivity testing results (minimum inhibitory concentrations, MICs) were interpreted and reported according to breakpoints published in the CLSI document M100-S23. Subsequently, the same samples were also analyzed by the clinical specimen-direct LAMP as previously reported [12]. Briefly, clinical samples were boiled and centrifuged, then supernatants were used as templates for the LAMP assay. Reactions proceeded at 65 °C for 20 min.

### Statistical analysis

Data are presented as frequencies with percentages for categorical variables, and median with interquartile range (IQR) for continuous variables. Baseline characteristics between groups were compared using Wilcoxon’s rank-sum test or chi-squared test. The primary outcome, which was the rate of CRAB acquisition between each period, was evaluated using Cox proportional hazard model with factors including period, age, sex, intubation, diabetes mellitus, and hypertension. In addition, we performed a multivariable Cox regression with multiple imputation of missing variables. The hazard ratio (HR) was shown with a 95% confidence interval (CI). As a secondary outcome, we used the multivariable Cox proportional hazard model including factors of medical interventions and antibiotic exposure in the intervention period. Risk factors associated with CRAB carriage on admission were analyzed using multivariable logistic regression since timing did not influence the results. All statistical analyses were performed using R version 3.4.1 (R Foundation for Statistical Computing, Vienna, Austria). All the tests were two-sided, and *p* < 0.05 was considered statistically significant.

## Results

### Characteristics of study participants during ICU stay

**Fig. 1:**
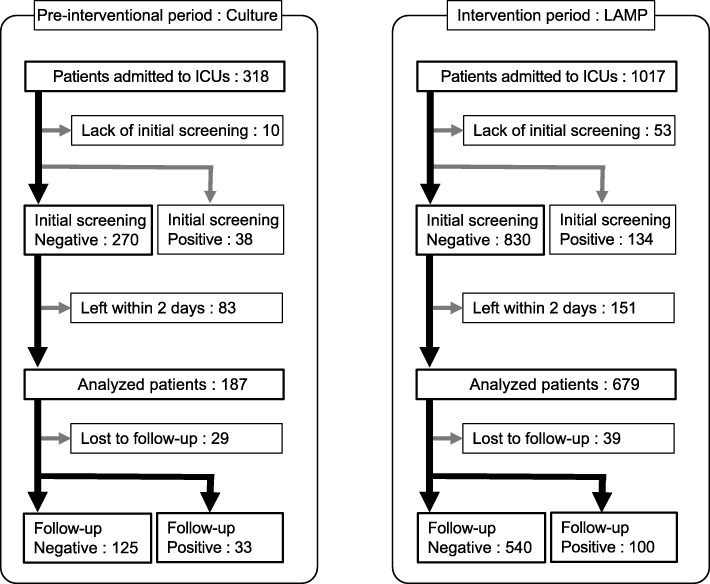
Flowchart for patient selection in this study

A total of 318 patients in the pre-intervention period and 1017 patients in the intervention period stayed in either ICU during the study term. Throughout both periods, 172 patients (38 and 134 respectively) were excluded from analysis of nosocomial transmission because they were already colonized with CRAB upon admission. Risk factors for carriage on admission are shown in Table 1. History of hospitalization and history of being a positive carrier were significantly associated with the carriage on admission. Patients who stayed less than 2 days, or whose initial screening was not appropriately implemented were excluded from calculation of acquisition rate. Overall, 866 patients (pre-intervention period: 187, intervention period: 679) were analyzed in this study (Fig. 1). We compared characteristics of the participating patients in each term (Table 2). Variables found to be statistically different between the two periods included diabetes mellitus, hypertension, infectious diseases.

**Table 1:** Risk factors associated with carriage of carbapenem-resistant *Acinetobacter baumannii* (CRAB) on admission to ICUs

Characteristics	Odds Ratio (95% CI)	*P*
Female Sex	1.02 (0.68–1.53)	0.93
Age	1.02 (1.00–1.03)	0.78
History of hospitalization within 90 days	2.85 (1.91–4.26)	< 0.001
History of CRAB-positive within 90 days	60.8 (29.7–125)	< 0.001
Circulatory disease	1.20 (0.73–1.97)	0.58
Chronic kidney disease	1.28 (0.78–2.10)	0.38
Gastrointestinal disease	1.64 (0.80–3.37)	0.18
Chronic respiratory disease	1.35 (0.80–2.30)	0.26
Hepatobiliary disease	0.89 (0.32–2.45)	0.82
Cerebral vascular disease	0.77 (0.30–1.96)	0.58
Malignancy (hematologic malignancies and solid tumor)	0.75 0.45–1.27)	0.30
Hypertension	0.85 (0.52–1.39)	0.51
Diabetes mellitus	0.78 (0.45–1.34)	0.37
Autoimmune disease	1.65 (0.65–4.16)	0.29

*CI* confidence interval

**Table 2:** Characteristics of patients eligible for this study

Characteristics	Pre-interventional Period (*n* = 187, %)	Interventional Period (*n* = 679, %)	Total (*n* = 866, %)	*P*
Female Sex	93 (49.7%)	315 (46.4%)	408 (47.1%)	0.42
Age, median years (IQR)	66 (55, 78)	64 (54, 77)	65 (54, 77)	0.46
History of hospitalization within 90 days	57 (30.5%)	209 (30.8%)	266 (30.7%)	0.94
History of CRAB positive within 90 days	5 (2.7%)	11 (1.6%)	16 (1.8%)	0.34
Duration of ICU stay, median days (IQR)	5 (3.5, 9)	6 (4, 10)	6 (4, 10)	0.124
Intubation	132 (70.6%)	460 (67.8%)	592 (68.4%)	0.48
Circulatory disease	38 (20.3%)	115 (16.9%)	153 (17.7%)	0.28
Chronic kidney disease	34 (18.2%)	123 (18.1%)	157 (18.1%)	0.98
Gastrointestinal disease	5 (2.7%)	43 (6.3%)	48 (5.5%)	0.053
Chronic respiratory disease	28 (15.0%)	87 (12.8%)	115 (13.3%)	0.44
Hepatobiliary disease	10 (5.3%)	26 (3.8%)	36 (4.2%)	0.36
Cerebral vascular disease	8 (4.3%)	55 (8.1%)	63 (7.3%)	0.075
Malignancy (hematologic malignancies and solid tumor)	41 (21.9%)	188 (27.7%)	229 (26.4%)	0.114
Hypertension	85 (45.5%)	225 (33.1%)	310 (35.8%)	0.002
Diabetes mellitus	62 (33.2%)	160 (23.6%)	222 (25.6%)	0.008
Autoimmune diseases	9 (4.8%)	28 (4.1%)	37 (4.3%)	0.68
Infectious diseases	62 (33.2%)	167 (24.6%)	229 (26.4%)	0.019

*IQR* interquartile range

### Transmission rate of CRAB in ICUs

A total of 34 and 100 patients obtained CRAB during the study periods, respectively. The transmission rate during ICU stay was 35.2 per 1000 patient-days in the pre-intervention period, compared with 20.9 per 1000 patient-days in the intervention period. The calculated hazard ratio of CRAB transmission was 0.65 (95% CI, 0.44–0.97) using the multivariable Cox proportional hazard analysis, implying rapid intervention successfully reduced 35% of nosocomial transmission (unadjusted *P* = 0.024, adjusted *P* = 0.033). Given 68 patients (29 and 39 respectively) had some missing values, we performed multivariable Cox regression with multiple imputation of missing variables. As a result, we could also observe a similar HR = 0.66 [95% CI, 0.44, 0.97; *P* = 0.034]. In summary, rapid intervention based on our LAMP assay contributed to reduced nosocomial transmission in ICUs.

### Risk factors for CRAB acquisition during ICU stay

To further confirm risk factors related to acquisition of CRAB, medical interventions being provided as well as antimicrobial exposures were analyzed as possible confounding factors (Table 3). Among all antibiotics, exposure to carbapenems significantly escalated the acquisition rate. Exposure to other antibiotics, as well as all the medical interventions analyzed including intubation, were not associated with CRAB acquisition.

**Table 3:** Risk factors associated with carbapenem-resistant *Acinetobacter baumannii* (CRAB) acquisition during hospitalization

Characteristics	Hazard Ratio (95% CI)	*p*
Medical intervention		
Intubation	1.23 (0.71–2.15)	0.46
Central venous catheter	0.69 (0.44–1.06)	0.091
Urinary Catheter	0.69 (0.39–1.23)	0.21
Steroid exposure	0.83 (0.53–1.29)	0.41
Antimicrobial exposure		
Carbapenems	2.54 (1.61–4.00)	< 0.0001
Quinolones	0.73 (0.40–1.34)	0.31
Penicillins	1.34 (0.87–2.08)	0.19
3rd and 4th generations of Cephalosporin	0.69 (0.45–1.06)	0.087
Aminoglycosides	1.59 (0.45–5.57)	0.47

*CI* confidence interval

### Performance of LAMP assay

The previously established clinical specimen-direct LAMP for *bla*_OXA-23_ CRAB positive testing [12] was used as a molecular diagnostic assay, and assay performance was evaluated throughout the study period. A total of 2517 clinical samples (tracheal aspirates: 861, rectal swabs; 1656) were obtained, resulting in a sensitivity of 99.1% (95% CI, 96.8–99.9%) and a specificity of 95.0% (95% CI, 94.0–95.8%) (Additional file 1: Table S1), when the gold standard was defined as the results based on culture. Our LAMP assay showed sufficiently high sensitivity and specificity to be used as a screening test in clinical settings.

## Discussion

The issue of CRAB transmission is critical in developing countries because it spreads unnoticed, and appropriate examinations cannot be performed because of a lack of resources [20–22]. The ICUs where this study was carried out were in an endemic situation. Overall, 13.5% of admitted patients tested positive for CRAB when entering the ICUs, a prevalence comparable with the cohort from Latibeaudiere et al. [6]. Positive patients were more likely to have histories of hospitalization and/or carriage, although other underlying diseases were not associated with carriage as shown in Table1. These results were similar to those of other studies [6, 23].

**Fig. 2:**
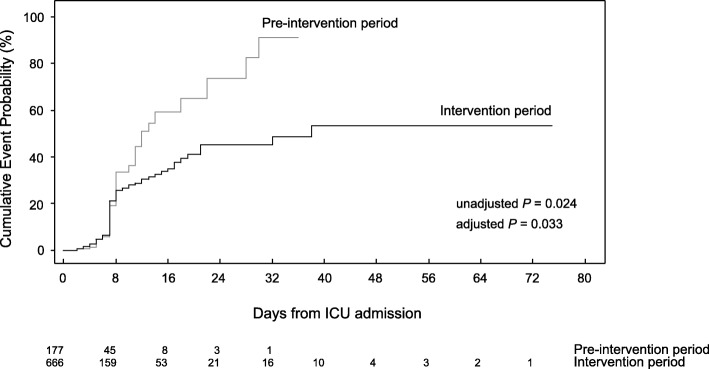
Kaplan-Meier curves representing cumulative incidence of acquiring carbapenem-resistant *Acinetobacter baumannii* during ICU stay. The hazard ratio (HR) was calculated by using the multivariable Cox proportional hazard analysis and HR of CRAB transmission was 0.65 (95% CI:0.44–0.97) in the interventional period compared with that in the pre-interventional period

Our rapid LAMP assay enabled us to implement earlier intervention for patients carrying CRAB. Using this approach, we achieved a 35% reduction in CRAB transmission in the ICUs compared with the use of the conventional culture method (Fig. 2). The difference during each period was in the time period for starting contact precautions. This difference alone contributed to significant reduction of CRAB nosocomial transmission. Although similar studies targeting methicillin-resistant *Staphylococcus aureus* have been reported [24–26], to the best of our knowledge clinical trials for CRAB transmission have never been performed.

The contamination rate of *A. baumannii* for environments as well as healthcare workers is higher than other multi-drug resistant organisms (MDROs) [27–31]. Once contaminated, an environment can be a CRAB reservoir for a long period of time since the pathogen can survive for more than several months under dry conditions [32]. Therefore, avoiding contamination is far more important with CRAB than other MDROs. We effectively reduced nosocomial transmission of CRAB in these ICUs because colonized patients were treated properly within a few hours after admission. However, nosocomial transmission could not be eradicated even after we implemented appropriate infection control measures immediately upon identifying a patient as a CRAB carrier. Part of the reason for this is that *A. baumannii* can be acquired directly from the environment, and there are some reports that this bacterium can even be transmitted through aerosols [33, 34]. These contaminated environments could be other origins of transmission.

We performed this study in ICUs where nosocomial transmission was most critical. The average duration of stay in the ICUs is generally shorter than general wards, with many patients leaving within 2 days’ duration before culture results can be obtained. In our study, 83 patients in the pre-intervention period and 151 patients in the intervention period left within 2 days, respectively. There were considerable numbers of such carriers among them and they could be generally unnoticed and considered as non-carriers. Using the rapid molecular LAMP assay method, we could implement appropriate precautions earlier than if culture was the only diagnostic test. Therefore, rapid intervention strategies might be more effective in ICU settings than on general wards.

Despite rapid implementation of preventive measures following the use of the LAMP assay, nosocomial transmission still occurred. We considered other potential medical interventions and/or medications that might be associated with acquisition of CRAB, and investigated risk factors for transmission during the intervention period as shown in Table 3. Exposure to carbapenems was associated with acquisition, a result comparable to other studies [35, 36]. We presume that carbapenems induce a selective pressure for acquisition of CRAB. Intubation, on the other hand, was not recognized as a risk factor in this cohort as other studies also mentioned that mechanical ventilation was not a risk for acquisition despite *A. baumannii* was major causative organisms of ventilator-associated pneumonia [5, 35].

To conduct this study, we adapted the original LAMP assay that targeted *bla*_OXA-23_-type without any DNA purification steps [12]. Consequently, this assay showed 99.1% sensitivity, although we targeted this genotype alone. This indicated the diagnostic LAMP test was applicable in clinical settings. Since the mechanism of acquiring drug resistance is becoming complicated, phenotyping methods using culture are often not sufficient for screening. However, PCR, the most popular of genetic analysis tools, is more expensive and laborious than our LAMP assay. The LAMP assay is applicable for such phenotyping experiments and LAMP can be performed without large equipment, demonstrating LAMP testing would be a strong tool to screen for resistant bacteria in resource-limited settings.

Our study could be affected by various factors, such as sample collection procedure, characteristics of ICU and anticipated patients, Hawthorne effect, and various compliances. The biggest bias of this study could be a before-after study design. However, we have compared the patients’ backgrounds and there were no significant differences in many variables and we have compensated in categories with significant differences. Furthermore, the pre-intervention period was shorter than the intervention period and was performed during a different period of the seasonal year. Therefore, we would not be able to remove any possible seasonal effect. For example, transmission of *Acinetobacter* species is more frequent in warm and humid climates [37, 38]. However, since the climate in Bangkok is ideal for *A. baumannii* throughout the year, the effect of the climate change would be minimized in our study settings. There are some more limitations to our study. First, this study was implemented in particular ICUs within a single center, and the results cannot be applied to any other clinical settings. In particular, the transmission rate was originally higher than the rate in high-income countries [39]. These background factors may impact the result. Second, we implemented this study using our original LAMP assay targeting *bla*_OXA-23_-type alone, although the efficacy had been confirmed. It could be difficult to perform similar research in other settings. Finally, some patients could not be screened or followed appropriately. This phenomenon was unavoidable, and represented 7.9%, of patients, which is comparable to previous studies [6, 24]. To account for this, we performed the multivariable Cox regression analysis with multiple imputation of missing variables.

In conclusion, we demonstrated the efficacy of using LAMP for rapid CRAB detection, allowing earlier precautions for CRAB carriers to be implemented in clinical settings. Several reports and guidelines recommended active surveillance for halting the spread of MDROs [3, 5, 7, 16]. Various novel techniques for bacterial identification are available, and these benefits are reported for treatment of infected patients [40, 41]. This study sheds light on the importance of implementing an immediate active screening test which can prompt earlier action than the conventional method. Rapid diagnostic tests, such as our LAMP assay, will play a critical role in avoiding transmission of CRAB in the future.

## Additional file

Additional file 1: Additional results of LAMP assay during study period. (DOCX 66 kb)

## Data Availability

The datasets used and/or analyzed during the current study are available from the corresponding author on reasonable request.

## Abbreviations

CRAB: Carbapenem-resistant *Acinetobacter baumannii*
ICU: Intensive care unit
LAMP: Loop-mediated isothermal amplification
MDROs: Multi-drug resistant organisms
