# Supplementary material for: Rapid screening and early precautions for carbapenem-resistant Acinetobacter baumannii carriers decreased nosocomial transmission in hospital settings: a quasi-experimental study
Source: Antimicrob Resist Infect Control. 2019 Jun 27;8:110. doi: 10.1186/s13756-019-0564-9 (PMC6598269; doi:10.1186/s13756-019-0564-9)
Supplement: Supplementary file 1 — Additional results of LAMP assay during study period. (DOCX 66 kb) [file 13756_2019_564_MOESM1_ESM.docx]

Table S1. Sensitivity and specificity of the loop-mediated isothermal amplification (LAMP) assay using bronchial aspirates and rectal swab samples.

| Specimens | Sensitivity [%] (95%CI) | Specificity [%] (95%CI) | PPV  (95%CI) | NPV  (95%CI) | PLR  (95%CI) | NLR  (95%CI) |
| --- | --- | --- | --- | --- | --- | --- |
| Bronchial aspirates | 100  (96.1 - 100) | 94.2  (92.4 – 95.7) | 0.65  (0.56 - 0.73) | 1.00  (0.99 - 1.00) | 17.22  (13.16 - 22.54) | 0  (0.00 - NA) |
| Rectal swab | 98.5  (94.7 – 99,8) | 95.3  (94.2 – 96.3) | 0.62  (0.55 - 0.69) | 1.00  (1.00 - 1.00) | 21.10  (17.05 - 26.13) | 0.02  (0.00 - 0.06) |
| Total | 99.1  (96.8 – 99.9) | 95.0  (94.0 – 95.8) | 0.63  (0.58 - 0.68) | 1.00  (1.00 - 1.00) | 19.64  (16.62 - 23.22) | 0.01  (0.00 - 0.04) |

*CI* confidence interval, *PPV* positive predictive value, *NPV* negative predictive value, *PLR* positive likelihood ratio, *NLR* Negative likelihood ratio.

The gold standard was defined as the results based on culture method to evaluate the efficacy of LAMP assay.
